# Frailty and risk of hospitalization from COVID-19 infection among older adults: evidence from the Dutch Lifelines COVID-19 Cohort study

**DOI:** 10.1007/s40520-022-02268-9

**Published:** 2022-10-16

**Authors:** Yinjie Zhu, Martine J. Sealy, Harriët Jager-Wittenaar, Jochen O. Mierau, Stephan J. L. Bakker, Gerjan J. Navis, Lifelines Corona Research Initiative, H. Marike Boezen, Jochen O. Mierau, H. Lude Franke, Jackie Dekens, Patrick Deelen, Pauline Lanting, Judith M. Vonk, Ilja Nolte, Anil P. S. Ori, Annique Claringbould, Floranne Boulogne, Marjolein X. L. Dijkema, Henry H. Wiersma, Robert Warmerdam, Soesma A. Jankipersadsing, Irene van Blokland, Geertruida H. de Bock, Judith G. M. Rosmalen, Cisca Wijmenga

**Affiliations:** 1 Division of Nephrology, Department of Internal Medicine, University of Groningen, University Medical Center Groningen, Hanzeplein 1, 9713GZ Groningen, The Netherlands; 2 Research Group Healthy Ageing, Allied Health Care and Nursing, Hanze University of Applied Sciences, 9714 CA Groningen, The Netherlands; 3 FAITH Research, Petrus Driessenstraat 3, 9714 CA Groningen, The Netherlands; 4 Department of Oral and Maxillofacial Surgery, University of Groningen, University Medical Center Groningen, Hanzeplein 1, 9713 GZ Groningen, The Netherlands; 5 Department of Economics, Econometrics and Finance, Faculty of Economics and Business, University of Groningen, University complex, 9747 AJ Groningen, The Netherlands; 6 Lifelines Cohort Study and Biobank, Groningen, The Netherlands; 7 Team Strategy and External Relations, University of Groningen, University Medical Center Groningen, Hanzeplein 1, 9713 GZ Groningen, The Netherlands

**Keywords:** Frailty, Groningen Frailty Indicator, COVID-19, Coronavirus

## Abstract

**Background:**

Frailty is associated with COVID-19 severity in clinical settings. No general population-based studies on the association between actual frailty status and COVID-19 hospitalization are available.

**Aims:**

To investigate the association between frailty and the risk of COVID-19 hospitalization once infected.

**Methods:**

440 older adults who participated in the Lifelines COVID-19 Cohort study in the Northern Netherlands and reported positive COVID-19 testing results (54.2% women, age 70 ± 4 years in 2021) were included in the analyses. COVID-19 hospitalization status was self-reported. The Groningen Frailty Indicator (GFI) was derived from 15 self-reported questionnaire items related to daily activities, health problems, and psychosocial functioning, with a score ≥ 4 indicating frailty. Both frailty and COVID-19 hospitalization were assessed in the same period. Poisson regression models with robust standard errors were used to analyze the associations between frailty and COVID-19 hospitalization.

**Results:**

Of 440 older adults included, 42 were hospitalized because of COVID-19 infection. After adjusting for sociodemographic and lifestyle factors, a higher risk of COVID-19 hospitalization was observed for frail individuals (risk ratio (RR) [95% CI] 1.97 [1.06–3.67]) compared to those classified as non-frail.

**Discussion:**

Frailty was positively associated with COVID-19 hospitalization once infected, independent of sociodemographic and lifestyle factors. Future research on frailty and COVID-19 should consider biomarkers of aging and frailty to understand the pathophysiological mechanisms and manifestations between frailty and COVID-19 outcomes.

**Conclusions:**

Frailty was positively associated with the risk of hospitalization among older adults that were infected with COVID-19. Public health strategies for frailty prevention in older adults need to be advocated, as it is helpful to reduce the burden of the healthcare system, particularly during a pandemic like COVID-19.

**Supplementary Information:**

The online version contains supplementary material available at 10.1007/s40520-022-02268-9.

## Introduction

Frailty is an age-related degeneration of several system organs that reflects the state of decreased reserve capacity and increased vulnerability to stressors [1] accompanied by multidimensional loss of energy, physical ability, cognition, and health [2]. Another concept has defined frailty as a dynamic state affecting an individual who experiences losses in one or more domains of the human functioning [3]. Despite the evolving and debatable conceptualization of frailty, adverse outcomes have been associated with frailty, including hospitalization, admission to long-term care, and mortality [4], which yield additional healthcare costs and extra burden for the healthcare system [5]. In addition, while age is a well-documented risk factor for COVID-19 outcomes, identifying frailty status is clinically relevant in further prioritizing vulnerable older individuals for the intensive care unit admission [4, 6].

Several systematic reviews and meta-analyses have indicated that frailty is an independent risk factor for COVID-19 severity and mortality. However, studies included in these reviews are only based on in-hospital patients, nursing homes, or long-term care units [7–10]. The association between frailty and COVID-19 severity has rarely been investigated in community-dwelling older adults. To our knowledge, Petermann-Rocha et al. were the first to report the association between frailty and risk of hospitalization or death from COVID-19 in a community-based study [11]. While they found that frailty was positively associated with severe COVID-19 infection, a significant limitation was that frailty was measured between 10 and 14 years prior to the COVID-19 outcome [11]. As frailty is dynamic and potentially preventable and reversible [12], their results should be carefully interpreted because the effect of frailty could be under- or over-estimated [11]. Therefore, more evidence for the association between the actual frailty status and COVID-19 outcomes is needed in community-dwelling older adults.

The Lifelines COVID-19 Cohort study assessed frailty status and COVID-19 outcomes around the same period and is linked to the Lifelines Biobank prospective cohort with a rich data background for its participants. This study aimed to investigate the association between frailty measured from the Groningen Frailty Indicator (GFI) and the risk of COVID-19 hospitalization in older adults infected with COVID-19 and participating in the Lifelines COVID-19 Cohort study.

## Methods

### Study population

The Lifelines COVID-19 Cohort is a questionnaire-based additional study collecting data about COVID-19-related symptoms, current health issues, and societal impacts from participants recruited from the Lifelines Cohort study [13]. The Lifelines Cohort study is a multidisciplinary population-based cohort study of 152,728 adults living in The Netherlands. It employs a broad range of investigative procedures in assessing the biomedical, sociodemographic, behavioral, physical, and psychological factors that contribute to the general population’s health and disease. Before study entry, a signed informed consent form was obtained from each participant. Adult participants (≥ 18 years) were asked to complete several self-administered questionnaires regarding various aspects, including demographics, socio-economic status, lifestyle factors, and morbidities. The Lifelines study was conducted according to the principles of the Declaration of Helsinki and approved by the Medical Ethics Committee of the Institutional Review Board of the University Medical Center Groningen, The Netherlands (2007/152). A detailed description of the Lifelines Cohort study can be found elsewhere [14, 15].

**Fig. 1:**
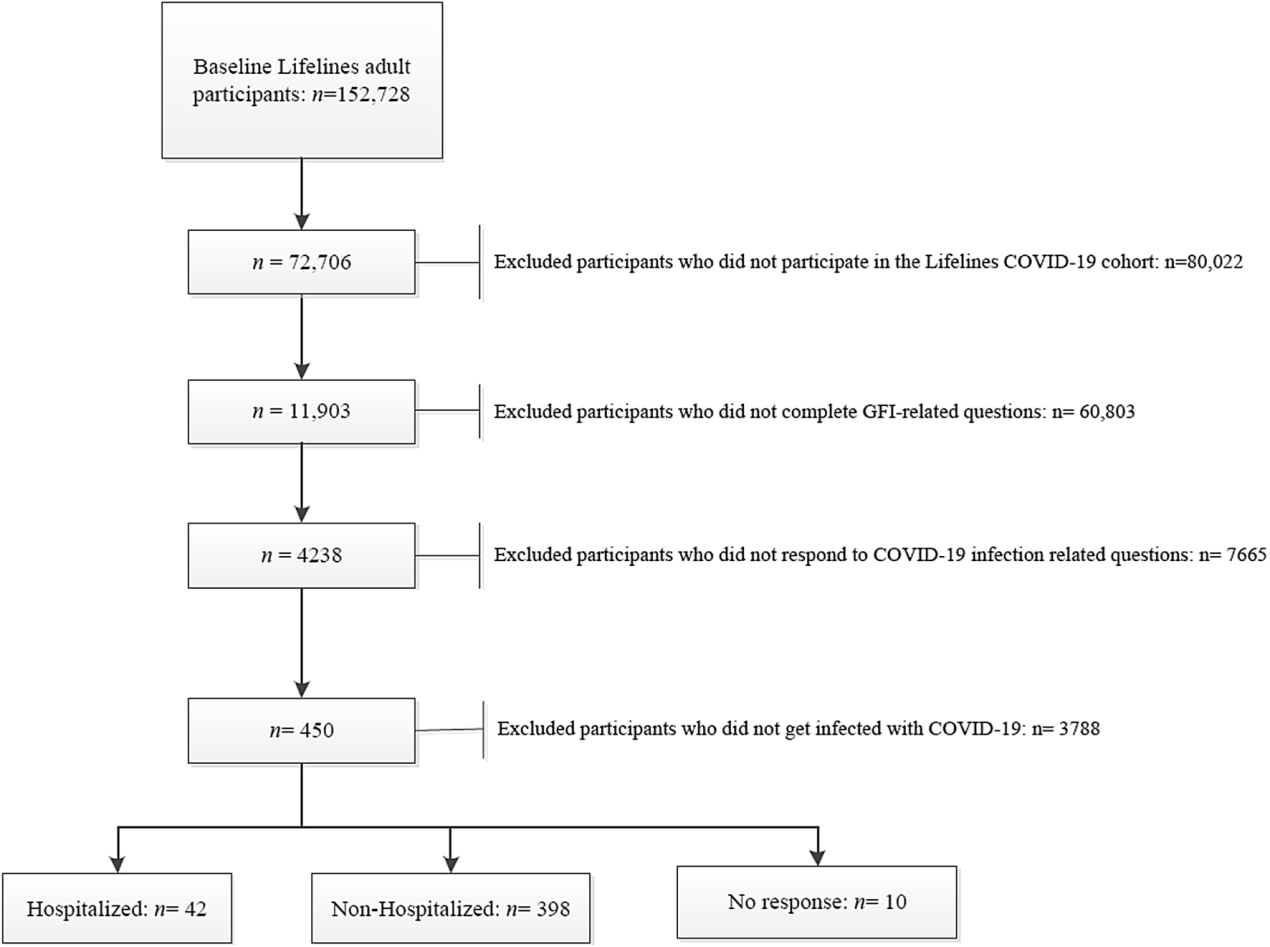
Study flowchart

The Lifelines COVID-19 Cohort study is developed based on a repeated COVID-19 questionnaire to identify genetic and environmental risk factors for COVID-19 and address the medical, social, and psychological aspects of the pandemic. Questions regarding frailty were only sent to participants aged ≥ 65 years. A detailed description of the Lifelines COVID-19 Cohort study and the COVID-19 questionnaire can be found elsewhere [13]. Questionnaires were sent on a (bi)weekly basis starting in March 2020 and on a monthly basis starting July 2020. In total, 22 questionnaires were sent until the end of July 2021, resulting in 72,706 adult participants who responded to at least one of the questionnaires. In the current study, we have included individuals with complete data on frailty-related questions who were infected with COVID-19 and reported their COVID-19 hospitalization status, leaving 440 participants in this study (Fig. 1).

### COVID-19 hospitalization

COVID-19 hospitalization was derived from the Lifelines COVID-19 questionnaire and was coded as a binary variable. COVID-19 hospitalization was obtained by asking “have you been hospitalized because of COVID-19?”, and only the participants who were infected with COVID-19 were further coded for the hospitalization status. COVID-19 infection was coded as one if the participant answered “yes” to the question “do you have or have you had a coronavirus/covid-19 infection?” or “positive” on the question “what were the results of your coronavirus (COVID-19) test?”. The 22 questionnaires were condensed into a single observation per individual, indicating for each question whether the individual had at any point answered “yes” or “positive” during the study period (March 2020 to July 2021). As COVID-19 hospitalization was reported among those infected with COVID-19, COVID-19 hospitalization is considered a proxy for the progress of COVID-19 infection.

### Groningen Frailty Indicator (GFI)

The GFI has been validated using baseline data from Lifelines [16, 17]. The original GFI instrument consists of 15 self-reported questionnaire items reflecting physical, cognitive, social, and psychological functions. The detailed version of the items and scoring method can be found elsewhere [18]. In short, individuals with and without impairment on each item score one and zero, respectively, resulting in GFI scores ranging from 0 to 15 by summing up the score of each item. A higher GFI score indicates a higher degree of functional impairment, with a score of four or higher representing frailty [17].

In the current study, GFI items were derived from the Lifelines COVID-19 questionnaire to operationalize the original GFI items, with nine items of the original GFI instrument and six comparable items that are identical to the original items (Supplementary Table S1 and Description S1). The application of the GFI in Lifelines COVID-19 data is described in detail elsewhere [19]. In accordance with the COVID-19 hospitalization variable, the GFI items were condensed into a single observation per individual during the study period and subsequently summed up with a GFI score of ≥ 4, indicating frailty. Frailty was included in the analyses as a binary variable.

### Covariates

Age was derived from birth years and the last COVID-19 questionnaire included in this study (July 2021). Educational level and presence of chronic diseases were derived from the baseline assessment of the Lifelines Cohort study because they are considered stable covariates and could not be derived from the COVID-19 questionnaire. Education level was assessed by self-reported questionnaires and was coded as categorical variables. The highest education level was categorized according to the International Standard Classification of Education (ISCED): (1) low (level 0, 1, or 2); (2) middle (level 3 or 4); and (3) high (level 5 or 6) [20]. The presence of chronic diseases within the cardiovascular, endocrinological, and renal domains was scored according to the 10th edition of the International Statistical Classification of Diseases and Related Health Problems (ICD-10) and was in line with the study by Dekker et al. [21].

Three self-reported lifestyle factors, i.e., smoking status, alcohol use, and physical activity, were derived from the first six Lifelines COVID-19 questionnaires due to data availability and further categorized into binary variables. Smoking status was derived from the question, “have you smoked in the past seven days” and a participant was considered smoking when “yes” was reported in at least one of the questionnaires. Alcohol use was derived from the question, “On average, how many glasses of alcohol per day have you used in the last seven days?”. Subsequently, the weekly alcohol use was calculated by multiplying seven by the reported daily alcohol use. Excessive drinking behavior was defined as consuming more than 21 drinks (male) or 14 drinks (female) per week [22]. A participant was defined as having excessive drinking behavior when the criteria of excessive drinking were met in at least one of the questionnaires. Physical activity was derived from the question, “in the last seven days, how many minutes of (moderately) intense activity did you do (e.g., walking, biking, or running)?”. The recommended moderate-to-vigorous physical activity (MVPA) is at least 150 min per week [23]. A participant was considered physically inactive if the reported MVPA was below 150 min per week in at least one of the questionnaires.

The average self-reported body weight was calculated from the first six Lifelines COVID-19 questionnaires. Height was measured objectively from the baseline assessment of the Lifelines Cohort study. Body mass index (BMI) was calculated as the average self-reported body weight (kg) divided by height squared (m^2^). The BMI was additionally categorized into suboptimal (BMI < 23 kg/m^2^), optimal (23 ≤ BMI < 30 kg/m^2^), and excess weight (BMI ≥ 30 kg/m^2^), given that the study population was ≥ 65 years old [24].

### Statistical analyses

Descriptive characteristics are presented as means with standard deviations (SD) for quantitative variables and percentages for categorical variables, broken down by frail and non-frail groups. Poisson regression models with robust standard errors were used to analyze the association between frailty and COVID-19 hospitalization. The results are reported as risk ratios (RRs) with 95% confidence intervals (CIs). Poisson regression models with robust standard errors were used because they provide RR estimates instead of odds ratios, which are easier to interpret [25, 26].

We built three models including an increasing number of covariates: model 1 (minimally adjusted), adjusted by age and sex; model 2, as per model 1 but also included education level; model 3, as per model 2 but also included three lifestyle factors (smoking, excessive drinking, and MVPA). Additional analyses (models 4, 5 and 6) were performed to investigate whether the association between frailty and COVID-19 was explained by the presence of chronic diseases or BMI. These models included covariates in model 3 but additionally included the presence of chronic diseases or/and BMI. All these covariates were selected because they have previously been recognized as being associated with the prognosis of COVID-19 as well as being associated with frailty status and may, therefore, potentially confound the relationship between frailty and COVID-19 hospitalization. We also analyzed the association between GFI as a continuous variable and COVID-19 hospitalization, adjusting for the same models mentioned above.

All statistical analyses were performed using Stata 13 (StataCorp, Texas, USA).

## Results

The characteristics of the total study population and stratified according to frailty status are shown in Table 1. Out of the 440 infected older adults who reported COVID-19 hospitalization status and GFI items completely, 157 (35.7%) participants were frail during the study period according to GFI, and 42 (9.5%) of them were hospitalized because of COVID-19 infection (Fig. 1). The mean age was 70 years, with 54.2% of the population being male. The majority of the population was Caucasian (99.1%). Nearly half of the participants had a low education level (46.2%), followed by middle (32.1%) and high (21.7%) education levels. The mean (SD) BMI was 26.6 ± 3.7 kg/m^2^, with 70.3% of the participants having an optimal BMI. The prevalence of smoking, being physically inactive, and excessive drinking were 4.2%, 75.9%, and 8.0%, respectively.

**Table 1:** Population characteristics

	Total	Frail (GFI ≥ 4)	Non-frail (GFI < 4)	*p*
Total, *n*	440	157	283	
Hospitalization, *n* (%)	42 (9.5)	22 (14.0)	20 (7.1)	0.02
Age in 2021, years	70 ± 4	70 ± 4	70 ± 4	0.9
Sex, female%	54.2	60.6	50.7	0.04
Education level, %				
Low	46.2	51.0	43.1	0.2
Middle	32.1	29.1	33.7	
High	21.7	19.0	23.3	
Ethnicity, Caucasian %	99.1	100	98.6	0.1
BMI, kg/m^2^	26.6 ± 3.7	27.4 ± 4.0	26.2 ± 3.4	< 0.001
Suboptimal, %	13.7	12.4	14.4	0.06
Optimal, %	70.3	66.0	72.7	
Excess weight, %	16.0	21.6	13.0	
Smoking, yes %	4.2	4.0	4.3	0.8
MVPA, < 150 min/w %	75.9	86.3	70.1	< 0.001
Excessive drinking, %	8.0	11.2	6.3	0.08
Chronic diseases, %				
CVD	35.3	38.1	33.8	0.4
Renal diseases	0.7	0.6	0.7	0.9
Endocrinological diseases	14.2	20.6	10.7	0.004

Excessive drinking is defined as consuming more than 21 drinks per week for men and more than 14 drinks per week for women

*MVPA* moderate-to-vigorous physical activity, *BMI* body mass index, *CVD* cardiovascular diseases

Compared to non-frail individuals, frail individuals had a higher prevalence of COVID-19 hospitalization (frail vs. non-frail: 14.0% vs. 7.1%), a higher BMI (frail vs. non-frail: 27.4 ± 4.0 kg/m^2^ vs. 26.2 ± 3.4 kg/m^2^), a higher prevalence of endocrinological diseases (frail vs. non-frail: 20.6% vs. 10.7%), and were more likely to be female (frail vs. non-frail: 60.6% vs. 50.7%) and physically inactive (frail vs. non-frail: 86.3% vs. 70.1%) (*p* < 0.05 for all, Table 1). In addition, frail individuals seemed to be less educated and more likely to drink excessively and have CVD (ns, Table 1).

Results on the association between frailty and COVID-19 hospitalization are presented in Table 2. Compared to non-frail individuals, being frail was associated with 2.09 times [95% CI 1.19–3.67] higher risk of COVID-19 hospitalization (model 1, Table 2). The association attenuated slightly but remained when additionally adjusting both for education level and lifestyle factors (frail vs. non-frail (ref) RR [95% CI]:1.97 [1.11–3.49] and 1.97 [1.06–3.67] in model 2 and model 3, Table 2). In addition, when the presence of chronic diseases and/or BMI was further included in models, frailty became borderline significantly associated with COVID-19 hospitalization (frail vs. non-frail (ref) RR [95% CI] 1.80 [0.96–3.39] in model 6, Table 2).

**Table 2:** Association between Groningen Frailty Indicator (GFI) and COVID-19 hospitalization^a^

	Model 1		Model 2		Model 3		Model 4		Model 5			Model 6	
	RR (95% CI)	*p*	RR (95% CI)	*p*	RR (95% CI)	*p*	RR (95% CI)	*p*		RR (95% CI)	*p*	RR (95% CI)	*p*
Frail (GFI ≥ 4)	2.09 (1.19–3.67)	0.01	1.97 (1.11–3.49)	0.02	1.97 (1.06–3.67)	0.03	1.87 (1.00–3.49)	0.05		1.85 (0.99–3.48)	0.06	1.80 (0.96–3.39)	0.07
Non-frail (Ref.; GFI < 4)	1		1		1		1			1		1	

^a^Data presented as RRs with their 95% CIs using Poisson regression analyses. Non-frail individuals were the reference group (“Ref”) based on GFI. Model 1, adjusted by age and sex; model 2, as model 1 but also included education level; model 3, as model 2 but also included lifestyle factors (smoking, alcohol use, and physical activity); model 4, as model 3 but also included the presence of chronic diseases; model 5, as model 3 but also included BMI; model 6, as model 3 but also included the presence of chronic diseases and BMI

After repeating models 1 to 3 treating GFI as a continuous variable, the RR was 1.16 (95% CI 1.03–1.30, model 3, Table 3) per 1 score increase in GFI. Further adjustments for the presence of chronic diseases and BMI only slightly weakened the association, with the RR decreasing to 1.14 (95% CI 1.01–1.28, model 6, Supplementary Table S3) per 1 score increase in GFI.

**Table 3:** Association between Groningen Frailty Indicator (GFI) as a continuous variable and COVID-19 hospitalization^a^

	Model 1		Model 2		Model 3		Model 4		Model 5		Model 6	
	RR (95% CI)	*p*	RR (95% CI)	*p*	RR (95% CI)	*p*	RR (95% CI)	*p*	RR (95% CI)	*p*	RR (95% CI)	*p*
GFI	1.17 (1.06–1.29)	0.002	1.15 (1.04–1.27)	0.008	1.16 (1.03–1.30)	0.02	1.14 (1.01–1.28)	0.03	1.15 (1.01–1.29)	0.03	1.14 (1.01–1.28)	0.04

^a^Data presented as RRs with their 95% CIs using Poisson regression analyses. Model 1, adjusted by age and sex; model 2, as model 1 but also included education level; model 3, as model 2 but also included lifestyle factors (smoking, alcohol use, and physical activity); model 4, as model 3 but also included the presence of chronic diseases; model 5, as model 3 but also included BMI; model 6, as model 3 but also included the presence of chronic diseases and BMI

## Discussion

In a group of older adults infected with COVID-19, we demonstrated that frailty was positively associated with the risk of COVID-19 hospitalization. Accounting for age, sex, education level, and lifestyle factors did not fully explain the association.

Our finding that frailty is associated with an increased risk of COVID-19 hospitalization once infected is consistent with previous studies in clinical and community-based settings [8, 11]. Moreover, our results showed that frailty, as an indicator of biological aging, could be a risk factor for COVID-19 hospitalization, independent of chronological aging, which is in accordance with another study suggesting that both chronological aging and frailty are independently associated with COVID-19 mortality [27]. Nevertheless, the interaction between frailty and COVID-19 hospitalization is likely bidirectional, as with other health outcomes [4]. More specifically, frailty could be a risk factor for the progress of COVID-19 infection because of the presence of excess weight, chronic diseases, and impaired respiratory function [27]. While the inflammatory reaction caused by COVID-19 infection may also exacerbate metabolic stress and muscle catabolism, resulting in malnutrition and physical inactivity, which could also cause frailty [28, 29].

We found a positive association between frailty and COVID-19 hospitalization, independent of non-biomolecular factors, including sociodemographic and lifestyle factors. However, further adjustments of the presence of chronic diseases and BMI attenuated the association to borderline significant while the RR remained clinically relevant, which can be attributed to the power issue when using a cut-off for frailty and be further supported by our additional analyses when treating GFI as the continuous independent variable (Table 3). Moreover, as BMI and presence of chronic diseases are both risk factors for COVID-19 hospitalization [30], which could mediate the association between frailty and COVID-19 hospitalization to some extent. The presence of chronic diseases is also comparable to some items included in the GFI, so the borderline significant associations could also be attributed to multicollinearity [31].

Besides the non-biomolecular factors, we acknowledge that it is essential to understand pathophysiological mechanisms via addressing potential biomolecular markers of frailty and aging as an increasing amount of research has reported how these biomarkers could predict COVID-19 progress [6]. The biomarkers of frailty might also substantially mediate the association between frailty and COVID-19 hospitalization in our study and present as sensitive indicators for frailty, as we still observed a trend for positive association even after accounting for two related elements of frailty, i.e., the presence of chronic diseases and BMI. In addition, several biomarkers of frailty and aging have also been found to be related to COVID-19 progress, including elevated C-reactive protein, interleukin-6, lactate dehydrogenase, cortisol, and low vitamin D levels [6]. However, these fingerprints of frailty and aging biomarkers could only provide one aspect explaining why COVID-19 outcomes have occurred disproportionally in frail and old individuals. Nevertheless, despite the number of candidate biomarkers, a better understanding of multisystem dysregulation and how its related to declines in the resilience of frail individuals are still needed because the pathophysiological mechanisms underlying frailty and how it is translated to disease outcomes, including COVID-19 progress, yet remain to be clarified [1, 32].

Both frailty and COVID-19 status are strongly associated with aging, and they also share several common risk factors, including excess weight, malnutrition, and impaired respiratory function [4, 30, 33]. While chronological aging cannot be modified, frailty is a multidimensional and dynamic status that could potentially be reversed and prevented with lifestyle interventions (physical activity and nutritional strategies) targeting physical frailty as well as other interventions focusing on psychological and social domains of frailty in older adults [34, 35]. Thus, identifying frail individuals as a higher risk group and providing tailored prevention programs could help reduce the burden of the healthcare system during the COVID-19 pandemic and have beneficial public health impacts. However, as frailty is multifactorial involving a wide spectrum of sociodemographic, psychological, clinical, lifestyle-related, and biological factors, its treatment should be multidisciplinary and intersectoral, which makes the execution of frailty prevention programs only at a population level more complex and less feasible and comprehensive [32]. Therefore, an ecological approach with not only lifestyle interventions at the community level but also with the implementation of a comprehensive geriatric assessment identifying intrinsic modifiable factors for individualized intervention and care plans could be considered. Still, this requires reorganizing the healthcare system and public health policies, which remains a long-term and challenging institutional issue [1, 32]. A more feasible approach to incorporate and highlight frailty in this rapidly evolving and still ongoing pandemic would be to actively involve clinical frailty assessment based on multidimensional frailty and biological age, rather than the traditional approach to advanced age at hospitalization. In other words, starting with frailty assessment in clinical settings to better prepare for further aggravation and simultaneously designing an ecological approach to assess and prevent frailty.

This study has several strengths. First, we provided evidence of older individuals from a general population instead of clinical settings; thus, our results had less selection bias and could be generalized at a national level, or other populations share identical characteristics with the Dutch population. Second, the response rate to COVID-19 infection questions (35.6%) was higher compared to previous studies. The French population-based CONSTANCES COVID-19 cohort reported a response rate of 13.3% [36], and a few British population-based cohorts reported response rates ranging from 12.2 to 33.6% [37]. Third, this study assessed the frailty status and COVID-19 hospitalization across the same period. Given that frailty is dynamic and potentially reversible, this study design could better capture the relationship between frailty and COVID-19 status compared to the previous study that assessed frailty more than a decade before the COVID-19 pandemic [11]. Fourth, utilizing the lifestyle factors and BMI assessed during the pandemic allowed us to determine the influence of restricted lifestyle during the pandemic on the association between frailty and COVID-19 hospitalization. It is well-known that lifestyle factors have changed dramatically during the pandemic because of the confinement; therefore, the assessment of lifestyle factors before the pandemic would no longer be representative [38]. Last, the instrument used to assess frailty, i.e., the GFI, has been validated and considered suitable for predicting health outcomes in our population [16, 18].

However, this study also has some limitations. First, despite that frailty and most of the covariates were measured during the Lifelines COVID-19 Cohort study, education level and presence of chronic diseases included in the analyses were identified from baseline Lifelines data (between 7 and 13 years prior to the first COVID-19 questionnaire). However, the education level was unlikely to have changed from the baseline assessment, given that the current population had an average age of 61 ± 4 years (range 53–70 years) at baseline. The presence of chronic diseases was assessed comprehensively, combining subjective reports and objective measurements, so the status of chronic diseases was also unlikely to be altered. Therefore, the two covariates were unlikely to introduce substantial bias in the models. Additionally, the physical activity assessment from the COVID-19 questionnaire was only validated in previous research [39] but not in the Lifelines Cohort due to the lack of objective assessments. Second, reporting bias could occur as the COVID-19 outcome was based on self-administered questionnaires, as older individuals with frailty might be less willing to answer COVID-19 related questions. Therefore, our results may underestimate the magnitude of the association between frailty and COVID-19 progress. Moreover, we were not able to differentiate between the types of COVID-19 tests that were performed, such as PCR test, rapid antigen test, or antibody test. Hence, it may be possible that misreporting of infection status should be noted. Third, the GFI was calculated partially from the original GFI items and partially from questions that are comparable to GFI items. We are not assured if the operationalization of the original GFI could be used interchangeably, although a recently published study that applied the same operationalized GFI seems to provide evidence on the validation of the operationalization of GFI [19]. Fourth, as we condensed the 22 questionnaires into observation per participant and GFI items were not collected every week, no causal inference could be derived from the association as we could not differentiate the bidirectional effect between frailty and COVID-19 hospitalization, as frailty could be the risk factor and outcome of COVID-19 hospitalization through the study period. Fifth, as mentioned above, we could not capture any effect of biomarkers of frailty as they were not included in the Lifelines COVID-19 Cohort study design. Finally, the dataset cannot exclude the inference of COVID-19 mortality, as individuals who died from COVID-19 infection were not able to answer the questions about hospitalization anymore. Thus, there might be an underestimation of the number of individuals admitted to the hospital due to COVID-19 infection. As previous studies suggested that frail individuals were also more likely to suffer from COVID-19 mortality, we do not expect the COVID-19 mortality to reverse or attenuate our results.

## Conclusions

Frailty was positively associated with COVID-19 hospitalization in a group of older adults of a general population in The Netherlands. Future studies conducted among community-dwelling older adults need to consider more frailty biomarkers to better understand the pathophysiological mechanism between frailty and various outcomes. Public health policymakers could consider an ecological and multidimensional approach to prevent and reserve frailty to better prepare our population, particularly the aging population, for healthy aging and future hazards.

## Supplementary Information

Below is the link to the electronic supplementary material.

Supplementary file1 (DOCX 24 KB)

## Data Availability

The authors do not have the authority to share the data that support the findings of this study, due to Lifelines data access permissions, but any researchers can apply to use Lifelines data, including the variables used in this investigation. Information about access to Lifelines data is given on their website: https://www.lifelines.nl/researcher/how-to-apply.
