# Supplementary material for: Frailty and risk of hospitalization from COVID-19 infection among older adults: evidence from the Dutch Lifelines COVID-19 Cohort study
Source: Aging Clin Exp Res. 2022 Oct 16;34(11):2693–702. doi: 10.1007/s40520-022-02268-9 (PMC9572827; doi:10.1007/s40520-022-02268-9)
Supplement: Supplementary file 1 — Supplementary file1 (DOCX 24 KB) [file 40520_2022_2268_MOESM1_ESM.docx]

**Title: Frailty and risk of hospitalization from COVID-19 infection among older adults: evidence from the Dutch Lifelines COVID-19 Cohort study.**

Aging Clinical and Experimental Research

Yinjie Zhu ^1^, Martine J. Sealy, Harriët Jager-Wittenaar, Jochen O. Mierau, Stephan J. L. Bakker, Gerjan J. Navis, Lifelines Corona Research initiative

^1^ Division of Nephrology, Department of Internal Medicine, University of Groningen, University Medical Center Groningen, Hanzeplein 1, 9713GZ Groningen, Groningen, The Netherlands.

Correspondence: Yinjie Zhu; email: [y.zhu@umcg.nl](mailto:y.zhu@umcg.nl); mailing address: Division of Nephrology, Zusterhuis, UMCG/IM-MIT, 9713 GZ, Groningen, The Netherlands

Supplementary Information

[Supplementary Table S1. Items and scores of the GFI in the Lifelines Covid-19 questionnaire (from Sealy MJ et al.) 1](#_Toc113877102)

[Supplementary Description S1. Assessment of GFI. 2](#_Toc113877103)

# Supplementary Table S1. Items and scores of the GFI in the Lifelines Covid-19 questionnaire (from Sealy MJ et al.)

| **Item no.** | **GFI-items** | **GFI-items in Lifelines Covid-19 questionnaire** |
| --- | --- | --- |
| 1 | Can you independently perform the following activities without any help from someone else, possibly with the help of a cane, walker or wheelchair?  Get groceries and run errands  *Yes = 0; No = 1* | Can you independently perform the following activities without any help from someone else, possibly with the help of a cane, walker or wheelchair?  Get groceries and run errands  *Yes = 0; No = 1* |
| 2 | Can you independently perform the following activities without any help from someone else, possibly with the help of a cane, walker or wheelchair?  Get dressed/undressed  *Yes = 0; No = 1* | Can you independently perform the following activities without any help from someone else, possibly with the help of a cane, walker or wheelchair?  Get dressed/undressed  *Yes = 0; No = 1* |
| 3 | Can you independently perform the following activities without any help from someone else, possibly with the help of a cane, walker or wheelchair?  Move outdoors (around house, to neighbors)  *Yes = 0; No = 1* | Can you independently perform the following activities without any help from someone else, possibly with the help of a cane, walker or wheelchair?  Move outdoors (around house, to neighbors)  *Yes = 0; No = 1* |
| 4 | Can you independently perform the following activities without any help from someone else, possibly with the help of a cane, walker or wheelchair?  Go the toilet  *Yes = 0; No = 1* | Can you independently perform the following activities without any help from someone else, possibly with the help of a cane, walker or wheelchair?  Go the toilet  *Yes = 0; No = 1* |
| 5 | What score would give your physical fitness (from 0 to 10)  *0-6=1; 7-10 = 0* | What score would give your physical fitness (from 0 to 10)  *0-6=1; 7-10 = 0* |
| 6 | Do you have problems in everyday life due to poor vision?  *Yes = 1; No = 0* | Do you have problems in everyday life due to poor vision?  *Yes = 1; No= 0* |
| 7 | Do you have problems in everyday life due to poor hearing?  *Yes= 1; No = 0* | Do you have problems in everyday life due to poor hearing?  *Yes = 1; No = 0* |
| 8 | Have you lost a lot of weight in the past period without wanting to (6 kg in 6 months or 3 kg in one month)?  *Yes = 1; No = 0* | Have you lost a lot of weight in the past period without wanting to (6 kg in 6 months or 3 kg in one month)?  *Yes = 1; No = 0* |
| 9 | Do you take 4 or more different types of medicine?  *Yes = 1; No = 0* | Number of different medicines from the medication checklist.  *≥4 = 1; <4 = 0* |
| 10 | Do you have memory problems?  *Yes = 1; No/sometimes = 0* | Do you have memory problems?  *Yes = 1; No/sometimes = 0* |
| 11 | Do you ever experience emptiness around you?  *Sometimes/yes = 1; No = 0* | How often do you feel disconnected from others in the last 7 days?  *Sometimes/often = 1; Almost never/never = 0* |
| 12 | Do you ever miss people around you?  *Sometimes/yes = 1; No = 0* | How often did you feel alone in the past 7 days?  *Sometimes/often = 1; Almost never/never = 0* |
| 13 | Do you ever feel let down?  *Sometimes/yes = 1; No = 0* | How often did you feel let down in the past 7 days?  *Sometimes/often = 1; Almost never/never = 0* |

* Sealy MJ, van der Lucht F, van Munster BC, Krijnen WP, Hobbelen H, Barf HA, et al. Frailty among Older People during the First Wave of the COVID-19 Pandemic in The Netherlands. Int J Environ Res Public Health. 2022;19(6).

# Supplementary Description S1. Assessment of GFI.

GFI items listed in Supplementary Table S1 were collected from the 1^st^, 6^th^. 10^th^, 14^th^, 16^th^, 18^th^, and 21^st^ out of the 22 COVID-19 questionnaires included in this study, except for types of medication use that were asked separately in the COVID-19 questionnaire from the 1^st^ to 9^th^, 14^th^, and 22^nd^ of the 22 COVID-19 questionnaires included in this study. Subsequently, these variables were condensed into one observation per participant, and score one was assigned to the participant when they reported at least once the impaired GFI items from the questionnaires that asked these items.
